# Seasonal variation in the canopy color of temperate evergreen conifer forests

**DOI:** 10.1111/nph.17046

**Published:** 2020-12-01

**Authors:** Bijan Seyednasrollah, David R. Bowling, Rui Cheng, Barry A. Logan, Troy S. Magney, Christian Frankenberg, Julia C. Yang, Adam M. Young, Koen Hufkens, M. Altaf Arain, T. Andrew Black, Peter D. Blanken, Rosvel Bracho, Rachhpal Jassal, David Y. Hollinger, Beverly E. Law, Zoran Nesic, Andrew D. Richardson

**Affiliations:** ^1^ School of Informatics, Computing & Cyber Systems Northern Arizona University Flagstaff AZ 86011 USA; ^2^ Center for Ecosystem Science and Society Northern Arizona University Flagstaff AZ 86011 USA; ^3^ School of Biological Sciences University of Utah Salt Lake City UT 84112 USA; ^4^ Division of Geological and Planetary Sciences California Institute of Technology Pasadena CA 91125 USA; ^5^ Jet Propulsion Laboratory California Institute of Technology Pasadena CA 91125 USA; ^6^ Department of Biology Bowdoin College Brunswick ME 04011 USA; ^7^ Department of Plant Sciences University of California Davis Davis CA 95616 USA; ^8^ Computational & Applied Vegetation Ecology Lab Ghent University Ghent 9000 Belgium; ^9^ INRA UMR ISPA Villenave d’Ornon 75011 France; ^10^ School of Earth, Environment and Society and McMaster Center for Climate Change McMaster University Hamilton ON L8S 4K1 Canada; ^11^ Faculty of Land and Food Systems University of British Columbia Vancouver BC V6T 1Z4 Canada; ^12^ Department of Geography University of Colorado Boulder CO 80309 USA; ^13^ School of Forest Resources and Conservation University of Florida Gainesville FL 32611 USA; ^14^ Northern Research Station USDA Forest Service Durham NH 03824 USA; ^15^ College of Forestry Oregon State University Corvallis OR 97330 USA

**Keywords:** AmeriFlux, evergreen conifer, PhenoCam, phenology, PRI, seasonality, xanthophyll

## Abstract

- Evergreen conifer forests are the most prevalent land cover type in North America. Seasonal changes in the color of evergreen forest canopies have been documented with near‐surface remote sensing, but the physiological mechanisms underlying these changes, and the implications for photosynthetic uptake, have not been fully elucidated.
- Here, we integrate on‐the‐ground phenological observations, leaf‐level physiological measurements, near surface hyperspectral remote sensing and digital camera imagery, tower‐based CO_2_ flux measurements, and a predictive model to simulate seasonal canopy color dynamics.
- We show that seasonal changes in canopy color occur independently of new leaf production, but track changes in chlorophyll fluorescence, the photochemical reflectance index, and leaf pigmentation. We demonstrate that at winter‐dormant sites, seasonal changes in canopy color can be used to predict the onset of canopy‐level photosynthesis in spring, and its cessation in autumn. Finally, we parameterize a simple temperature‐based model to predict the seasonal cycle of canopy greenness, and we show that the model successfully simulates interannual variation in the timing of changes in canopy color.
- These results provide mechanistic insight into the factors driving seasonal changes in evergreen canopy color and provide opportunities to monitor and model seasonal variation in photosynthetic activity using color‐based vegetation indices.

## Introduction

Evergreen conifer forests are the dominant land cover type across much of the mid‐latitudes of North America (Pielou, 2011). These forests account for a substantial portion of continental‐scale CO_2_ uptake and carbon sequestration, influence water cycling and the ecohydrology of some of the continent's largest watersheds, and have major effects on land–atmosphere interactions that are relevant in the context of the global Earth system (Bonan, 2008). Therefore, they play a critical role in the response of the terrestrial biosphere to environmental change.

In cold climates, conifer forests are photosynthetically dormant during the winter months, despite maintaining foliage year‐round (Hänninen, 2016). Downregulation of photosynthetic capacity occurs in response to shorter and cooler days in autumn (Hollinger *et al*., 1999; Way & Montgomery, 2015; Gamon *et al*., 2016; Bowling *et al*., 2018). Likewise, upregulation of photosynthetic capacity is observed to occur in spring in response to increasing temperature (Tanja *et al*., 2003; Richardson *et al*., 2009b). However, incident solar radiation at temperate latitudes remains relatively high during winter, and thus the photosynthetic apparatus must be protected from excess absorbed energy to prevent damage (Demmig‐Adams & Adams, 1996). Conifers achieve winter photoprotection through thermal dissipation of excess absorbed excitation energy (Míguez *et al*., 2015). As reviewed by Verhoeven (2014) and Ensminger *et al*. (2006), this appears to involve both rearrangement of the photosynthetic apparatus—with the light harvesting centers essentially being converted into ‘energy dissipating centers’—and upregulation of the xanthophyll cycle.

Conventional remote sensing indices, which are based on the red‐edge transition between low visible‐wavelength reflectance and high near infrared‐wavelength reflectance (e.g. normalized difference vegetation index or NDVI; Tucker, 1979) typically fail to robustly detect seasonal changes in the carotenoid pigment pool (Magney *et al*., 2019) or photosynthetic activity (Balzarolo *et al*., 2016; Gamon *et al*., 2016) in evergreen conifer forests. Intriguingly, visible‐wavelength indices may offer improved potential for remote sensing of the seasonality of evergreen conifer photosynthesis (Wong & Gamon, 2015a). Gamon *et al*. (2016) presented a ‘chlorophyll/carotenoid index’ (CCI), calculated from bands 11 (531 nm) and 1 (645 nm) on NASA’s MODerate resolution Imaging Spectroradiometer (MODIS) satellite sensors. CCI performed better than NDVI at characterizing the seasonality of daily gross photosynthesis (or gross primary productivity, GPP, estimated from eddy covariance CO_2_ flux measurements) across three evergreen sites.

Other studies have shown that visible‐wavelength indices derived from digital camera (or ‘phenocam’; Richardson, 2019) imagery offer similar potential (Toomey *et al*., 2015), although it is uncertain whether any single color‐based index can robustly track seasonal changes in photosynthetic activity across different evergreen species mixtures, ecosystem types, or climate regimes. Richardson *et al*. (2009a) reported that a ‘green excess’ index mimicked the seasonal pattern of GPP at the conifer‐dominated Howland Forest. Results from the Niwot Ridge subalpine forest (Colorado, USA) suggested that seasonal variation in the light‐saturated rate of canopy photosynthesis, a proxy for photosynthetic capacity, was better correlated (*r* = 0.92 vs *r* = 0.76, respectively) with an index known as the green chromatic coordinate (*G*_cc_) than with the green–red vegetation index (GRVI), an approximation to CCI (Bowling *et al*., 2018). Most recently, Liu *et al*. (2020), reported that start‐of season (SOS) and end‐of‐season (EOS) transition dates derived from the red chromatic coordinate (*R*_cc_) better predicted seasonal transitions in evergreen conifer photosynthesis than transition dates derived from *G*_cc_. However, the seasonal trajectory of *R*_cc_ did not itself align with the seasonal trajectory of GPP, and day‐to‐day variation in *R*_cc_ was much higher than *G*_cc_. On top of uncertainties about which is the best index to use, mechanistic studies to understand the underlying physiological basis for the observed seasonal variation in canopy color have been lacking.

Upregulation of photosynthetic capacity in spring has been modeled as a function of air temperature (Tanja *et al*., 2003; Richardson *et al*., 2009b, 2019), and observational studies point to temperature as the key factor driving concurrent seasonal changes in evergreen conifer canopy color and photosynthesis (e.g. Bowling *et al*., 2018). Experimental warming has been shown to advance spring ‘green‐up’ transition dates, and delay autumn ‘green‐down’ transition dates (both derived from *G*_cc_) for mature black spruce trees (Richardson *et al*., 2018b). To date, the potential to model the seasonal trajectory of evergreen conifer canopy color as a function of temperature has not yet been investigated.

Here, using data from evergreen conifer sites across the PhenoCam Network, we explore the relationships between seasonal variation in canopy color and photosynthetic activity. We begin by using leaf‐level field data from three winter‐dormant sites (Howland Forest, Harvard Forest, and Niwot Ridge) to investigate the physiological mechanisms associated with seasonal changes in canopy color. Next, using data from 11 sites in the AmeriFlux and FLUXNET‐Canada networks, we conduct an analysis that links seasonal variation in *G*_cc_ and GRVI to the seasonality of GPP at the seven sites that are winter‐dormant. Finally, using a multi‐year time series of *G*_cc_ from 26 evergreen PhenoCam sites, we parameterize and test a temperature‐based model that simulates the seasonal trajectory of *G*_cc_, from winter dormancy to the summertime peak in activity and back to winter dormancy.

## Materials and Methods

### PhenoCam data

The PhenoCam Network uses digital repeat photography to track seasonal changes in canopy color at over 600 sites across North America and around the world (Richardson, 2019). Over 100 sites in the network include evergreen conifer vegetation, and the most recent curated PhenoCam data release (v.2; Seyednasrollah *et al*., 2019b) includes 265 site‐years of data for evergreen conifer sites. The PhenoCam processing workflow is fully described in previous publications (Richardson *et al*., 2018a; Seyednasrollah *et al*., 2019a); we summarize the key steps here.

Imagery used in the current study was obtained from cameras (NetCam SC IR; StarDot Technologies, Buena Park, CA, USA) deployed (see http://phenocam.sr.unh.edu/) and configured (https://khufkens.github.io/phenocam‐installation‐tool/) using a common protocol. Images were recorded from 04:00 to 22:00 (local time; images captured when the sun was less than 5° above the horizon were excluded), uploaded to the PhenoCam server, and processed to yield quantitative information about canopy color for a predefined region of interest (ROI) corresponding to the vegetation under study. We used only ROIs specific to evergreen needleleaf (EN) vegetation, while excluding exposed ground and other vegetation types, in this analysis.

For each image, the mean intensity (across the ROI) of the red, green, and blue (RGB) color channels was determined, and from these 8‐bit digital numbers (*R*_DN_, *G*_DN_, *B*_DN_) we calculated the *G*_cc_ and GRVI (Richardson *et al*., 2013b; Bowling *et al*., 2018) as:

(Eqn 1) $$G_{\text{cc}}=\frac{G_{\text{DN}}}{R_{\text{DN}}+G_{\text{DN}}+B_{\text{DN}}}$$

(Eqn 2) $$\text{GRVI}=\frac{G_{\text{DN}}‐R_{\text{DN}}}{G_{\text{DN}}+R_{\text{DN}}}$$

Following Richardson *et al*. (2018b), we visually inspected the imagery from each camera, and identified days on which the canopy was at least partially obscured by snow at midday. These days were excluded from analysis. To further minimize the effects of variation in lighting conditions (e.g. clouds, aerosols, and illumination geometry), we aggregated data from multiple images recorded each day to composite products at a 1‐ and 3‐d time step using the quantile‐based approach described by Sonnentag *et al*. (2012). We then used a LOESS‐based method to smooth the *G*_cc_ and GRVI time series, identify outliers, and extracted seasonal transition dates based on predefined thresholds (10%, 25%, and 50%) of the seasonal amplitude (Richardson *et al*., 2018a; Seyednasrollah *et al*., 2019a).

**Fig. 1:**
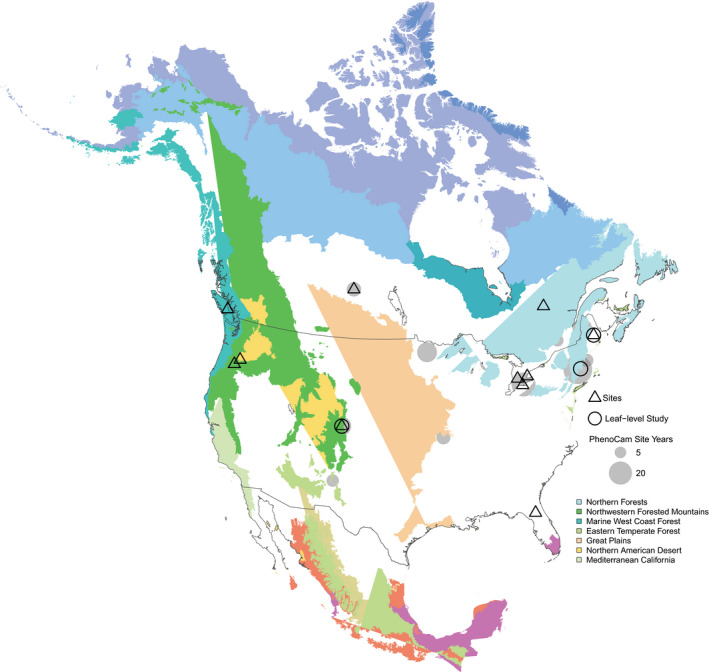
Distribution of the study sites used in the present analysis. PhenoCam site‐year counts, used in the modeling analysis, are aggregated to 1° × 1° grids.

All PhenoCam data used here (Fig. 1) are publicly available from the PhenoCam dataset v.2.0 (Seyednasrollah *et al*., 2019a) which is available for download from the ORNL DAAC (Seyednasrollah *et al*., 2019b).

### Leaf‐level observations and measurements

#### Howland Forest

The Howland Forest site (US‐Ho1, 45.2°N, 68.7 °W, elevation 60 m above sea level (asl) is located in a boreal‐northern hardwood transition forest about 50 km north of Bangor, ME, USA. Forest composition is dominated by the evergreen conifers, red spruce (*Picea rubens* Sarg., 44% of basal area) and eastern hemlock (*Tsuga canadensis* (L.) Carrière, 26% of basal area). Mean annual temperature (MAT) is 5.3°C and mean annual precipitation (MAP) is 1070 mm. Phenological observations (budburst) have been conducted at Howland Forest since 1990 and are ongoing. The mean aggregate date of budburst by the dominant coniferous species was estimated from visual observations made during weekly or twice‐weekly site visits (Richardson *et al*., 2009b). A phenocam was installed at Howland in 2007.

#### Harvard Forest

The Harvard Forest (US‐Ha1, 42.5 °N, 72.2°W, elevation 340 m asl) is a mixed temperate forest about 110 km west of Boston, MA, USA. Forest composition is dominated by the deciduous species red oak (*Quercus rubra* L., 36% of basal area) and red maple (*Acer rubrum* L., 22% of basal area), although mixed and pure stands of evergreen conifer species are found throughout the forest, for example eastern hemlock (13% of basal area) and white pine (*Pinus strobus* L., 6% of basal area). MAT is 6.6°C and MAP is 1070 mm. Field observations of phenology have been conducted at Harvard Forest since 1990 (Richardson & O’Keefe, 2009) and are ongoing, although observations of budburst for both white pine and eastern hemlock were discontinued in 2001. Phenocams were installed on the Hemlock tower (forest dominated by eastern hemlock) in 2010 and the Barn tower (mixed forest dominated by red oak and white pine) in 2011.

Leaf‐level physiological studies of white pine and eastern hemlock were initiated at Harvard Forest in mid‐winter 2015 and continued through late‐winter 2016. Needle samples were collected from three trees of each species in the vicinity of the Hemlock and Barn towers, monthly during the dormant season and every 2 weeks during spring and autumn. The chlorophyll fluorescence ratio *F*_v_ */F*_m_ was measured using a hand‐held Opti‐sciences OS‐30p fluorometer (Opti‐Sciences, Hudson, NH, USA) on needles (*n* = 20) that had been dark acclimated for a minimum of 15 min. Spectral reflectance (350–2500 nm) was measured using a FieldSpec 3 portable spectrometer (Analytical Spectral Devices, Boulder, CO, USA). The spectrometer was connected to a needle leaf clip (PP Systems, Amesbury, MA, USA), with illumination provided by a quartz tungsten halogen light source (Thorlabs SLS201L), referenced to a white Spectralon (Labsphere, North Sutton, NH, USA) standard. We calculated the photochemical reflectance index (PRI; Gamon *et al*., 1992, 1997) using reflectance at 531 and 570 nm (*ρ*_531_ and *ρ*_570_, respectively) as:

(Eqn 3) $$PRI=\frac{\rho_{531}‐\rho_{570}}{\rho_{531}+\rho_{570}}$$

#### Niwot Ridge

The subalpine Niwot Ridge site (US‐NR1, 40.0°N, 105.6°W, elevation 3050 m asl) is located about 25 km west of Boulder, CO, USA. Forest composition is dominated by the evergreen conifer species: subalpine fir (*Abies lasiocarpa* (Hook.) Nutt., 46% stem density), Engelmann spruce (*Picea engelmannii* Parry ex Engelm., 28% stem density), and lodgepole pine (*Pinus contorta* Dougl. ex. Loud., 26% stem density). MAT is 1.5°C and MAP is 800 mm, of which 65% typically falls as snow. A phenocam was first installed at Niwot Ridge in 2009.

We used leaf pigment data from Magney *et al*. (2019) to investigate relationships between changes in canopy color and leaf‐level pigment pools and ratios. Approximately monthly from June 2017 to June 2018, samples were collected from two lodgepole pine and three Engelmann spruce trees growing in close proximity to the tower, and flash‐frozen in liquid nitrogen. Pigments were extracted in acetone and analyzed by high‐performance liquid chromatography (HPLC; Bowling *et al*., 2018). Pigments measured included Chl*a* and Chl*b*, violaxanthin (V), antheraxanthin (A), zeaxanthin (Z), neoxanthin, lutein, and α‐ and β‐carotene.

Three of the trees sampled for pigments (two lodgepole pines and one Engelmann spruce) were in the field of view of the *niwot5* phenocam. We developed custom ROI masks and extracted canopy color information for each tree on the day on which pigment sampling occurred. We used these data to assess the linear correlations between color indices (*G*_cc_ and GRVI) and pigment contents (total chlorophyll, total carotenoids, and total xanthophyll cycle pigments), their ratios (chl : car, chl : xan), and xanthophyll cycle epoxidation state ([Z + A]/[V + Z + A]).

For further insight into relationships between pigments and seasonal changes in canopy color, we used hyperspectral reflectance measured from a two‐dimensional (2D) scanning telescope, PhotoSpec (Grossmann *et al*., 2018), mounted on top of the Niwot Ridge AmeriFlux tower (Cheng *et al*., 2020). PhotoSpec measures canopy reflectance (*ρ*_λ_) from 400 nm to 900 nm (1.2 nm full‐width‐half‐maximum) with a Flame‐S spectrometer (Ocean Optics Inc., Dunedin, FL, USA). The field of view of PhotoSpec is 0.7°. The narrow field of view allowed us to point the instrument at the individual trees that were sampled for pigment analysis. From the PhotoSpec data, we calculated a full suite of normalized difference reflectance indices (NDIs), using all possible combinations for *ρ*_λ1_ and *ρ*_λ2_, as in Eqn 4.

(Eqn 4) $$NDI\left(\lambda_{1},\lambda_{2}\right)=\frac{\rho_{\lambda 1}‐\rho_{\lambda 2}}{\rho_{\lambda 1}+\rho_{\lambda 2}}$$

### Eddy covariance measurements of forest–atmosphere exchange

To place seasonal changes in canopy color in the context of the seasonality of canopy photosynthesis, we used tower‐based eddy covariance measurements of forest–atmosphere CO_2_ fluxes (Baldocchi, 2008). We identified 11 evergreen sites in North America that had multiple years of flux data with overlapping PhenoCam data (Supporting Information Table S1). The eddy covariance measurements (net ecosystem exchange (NEE) of CO_2_, in µmol m^−2^ s^−1^) were obtained from the AmeriFlux (sites: US‐Ho1, US‐NR1, US‐Me2, US‐Me6, CA‐TP1, CA‐TP3, CA‐TP4, and CA‐Obs) and FLUXNET2015 (CA‐Qfo) archives, or directly from site PI (Ca‐Obs (most recent data), Ca‐Ca3, and US‐SP1).

Our workflow for processing the CO_2_ flux measurements is illustrated in Fig. S1. The flux data were evaluated to determine an appropriate friction velocity threshold (u*) to remove periods of low turbulence, and flux and meteorological data were then gap‐filled using the reddyproc package in R (Wutzler *et al*., 2018).

Gross primary productivity (GPP) was derived following Hollinger *et al*. (2004) by first estimating ecosystem respiration (*R*_eco_) from the average nighttime NEE over a moving 5‐d window, and then subtracting *R*_eco_ from NEE. The light response of GPP was characterized using a Michaelis–Menten approach (Eqn 5):

(Eqn 5) $$\text{GPP}=A_{\max}\times \text{PPFD}/\left(K_{m}+\text{PPFD}\right)$$

where PPFD is photosynthetic photon flux density (in µmol m^−2^ s^−1^), *A*_max_ is the theoretical maximum rate of photosynthesis, and *K*_m_ is the half‐saturation constant; *A*_max_ and *K*_m_ were fit parameters. The canopy‐level photosynthetic capacity index (Bowling *et al*., 2018), GPP_sat_, was calculated by evaluating the fit to Eqn 5 within each moving window, assuming full‐sun conditions (PPFD = 2000 µmol m^−2^ s^−1^).

The GPP_sat_ time series were used to determine the start and end of the photosynthetically active season (SOS and EOS, respectively, see Fig. S1). For each year, the time series of daily GPP_sat_ was smoothed with a LOESS filter, from which the annual maximum GPP_sat_ was determined. At winter dormant sites, the annual minimum of GPP_sat_ was zero (Fig. S1). Similar to how transition dates were determined from PhenoCam data, the GPP‐based SOS transition dates were determined as the day that the smoothed GPP_sat_ crossed the thresholds of 10, 25 and 50% (SOS10, SOS25, and SOS50) of the annual maximum GPP_sat_. EOS transition dates were similarly determined during the decrease of GPP_sat_ in autumn. Uncertainties on these transition dates were estimated by bootstrapping.

We evaluated the agreement between PhenoCam‐based transition dates and GPP‐based transition dates using Type II regression and correlation analysis. Because there are various ways to statistically aggregate (e.g. mean, median, 75^th^ and 90^th^ quantile) the *G*_cc_ and GRVI data from multiple images to a single 3‐d index value, and then to extract SOS and EOS transition dates from 3‐d data using different thresholds (10%, 25%, and 50%) of the seasonal amplitude (Richardson *et al*., 2018a), we evaluated all 12 possible combinations of these approaches for both *G*_cc_ and GRVI (Table S3, see later). We report results here for only one method each for *G*_cc_ and GRVI. We selected the ‘best’ method by identifying, for each index, the method that had the highest aggregate correlation (sum of SOS and EOS PhenoCam–GPP correlation coefficients) across all site‐years of data. For *G*_cc_, 3‐d values were calculated as the 90^th^ quantile of the index, calculated across all daytime images, and transition dates were determined based on the 25% threshold of the seasonal amplitude. For GRVI, 3‐d values were calculated as the mean value of the index, across all daytime images, and transition dates were determined based on the 50% threshold of the seasonal amplitude.

### Modeling the seasonal trajectory of canopy color

We developed a temperature‐driven model to simulate the seasonal trajectory of canopy greenness (cf. directly modeling SOS and EOS transition dates, e.g. Melaas *et al*., 2016; Richardson *et al*., 2019b), whereby day‐to‐day changes in greenness are driven by the preceding day's maximum (spring) or minimum (autumn) air temperature. The model was inspired by formulations previously presented to simulate the seasonality of developmental processes (Hänninen & Kramer, 2007; Hänninen, 2016), and seasonal upregulation and downregulation of photosynthetic capacity (Richardson *et al*., 2010b) of winter‐dormant conifers. Our Bayesian framework (Eqns (Eqn 6), (Eqn 7), (Eqn 8)) accommodates missing data and uncertainty in the observations, which makes it powerful for statistical inference from phenological time series.

(Eqn 6) $$Gcc_{s,t}∼N\left(\gamma_{s,t},\sigma^{2}\right)$$

(Eqn 7) $$\gamma_{s,t}=\min\left(\max\left(\gamma_{s,t‐1}+\Delta \gamma_{s,t},\gamma_{s,\min}\right),\gamma_{s,\max}\right)$$

(Eqn 8) $$\Delta \gamma_{s,t}=\left\{\begin{array}{c} \max\left(T_{\max,s,t}‐\theta_{s,1},0\right)\cdot \rho_{s,1}t<D\\ \min\left(T_{\min,s,t}‐\theta_{s,2},0\right)\cdot \rho_{s,2}t\geq D \end{array}\right)$$

Briefly, canopy greenness, *G*_cc_ (Eqn 6), for site *s* at time *t* is assumed to be distributed by a Gaussian distribution (*N*) with mean $\gamma_{s,t}$ and variance $\sigma^{2}.$ The state variable, $\gamma_{s,t}$ (Eqn 7), tracks daily changes in *G*_cc_ and is constrained to fall within the range defined by *γ_s_*_,min_ and *γ_s_*_,max_, which are estimated parameters. The daily change in that state variable, ∆$\gamma_{s,t}$ (Eqn 8), is prescribed as a function of air temperature. In spring, the increase in *G*_cc_ occurs as the product of the daily *T*_max_ above the threshold temperature $\theta_{s,1}$, multiplied by the temperature sensitivity factor, $\rho_{s,1}$. In autumn, the decrease in *G*_cc_ occurs as the product of the daily *T*_min_ below the threshold temperature $\theta_{s,2}$, multiplied by the temperature sensitivity factor, $\rho_{s,2}$. The timing of the transition from the spring phenology phase to the autumn phenology phase is determined by the parameter *D*, which is specified as a day of year.

The posterior distributions of the model parameters ($\theta_{s,1}$, $\theta_{s,2}$ $\rho_{s,1}$, $\rho_{s,2}$, and *D*) were fitted on a site‐by‐site basis for 26 evergreen conifer PhenoCam sites in North America (Table S2). We selected evergreen conifer sites with more than 4 yr of data in the PhenoCam data set v.2.0 (Seyednasrollah *et al*., 2019a) and where the seasonal signal‐to‐noise ratio was at least 20 : 1. We quantified ‘signal’ as the seasonal amplitude of *G*_cc_, and ‘noise’ as the mean absolute difference in *G*_cc_ across successive days (over which time phenological change is expected to be small). Daily minimum and maximum temperature data were obtained from Daymet (Thornton *et al*., 2016). A Gibbs sampling method (‘rjags’ package in R 4.0; Plummer, 2013) was used to fit the posterior distributions. For cross‐validation of an *n*‐year time series, the first *n* – 1 year were used for model calibration parameters (see Fig. S2), and the final year of data was used for out‐of‐sample validation. To evaluate the ability of the model to capture interannual variation in the timing of phenological shifts, SOS and EOS transition dates were estimated from the model as 50% of the seasonal amplitude of simulated *G*_cc_ and compared against the 50% seasonal amplitude transition dates described in section entitled ‘PhenoCam data’.

## Results

### Leaf‐level observations

#### Howland Forest

**Fig. 2:**
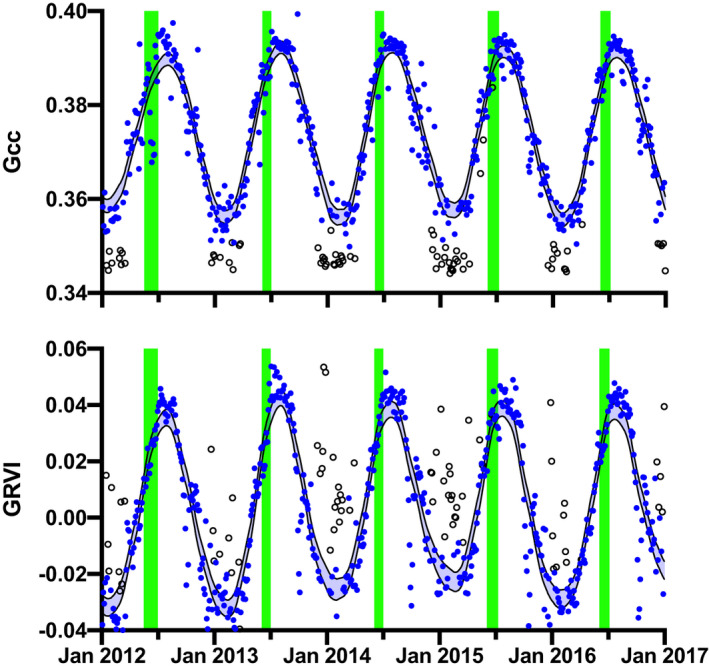
Seasonality of canopy color, as characterized by the green chromatic coordinate (*G*_cc_, upper panel) and green–red vegetation index (GRVI, lower panel), from PhenoCam imagery for Howland Forest. Filled blue symbols indicate 3‐d composite values from high‐frequency imagery; hollow symbols are data that have been screened because of snow on the evergreen canopy. Shaded blue bands indicate LOESS smoothing spline fit to the snow‐filtered data, ± 95% confidence interval. Vertical green bars indicate the period between conifer budburst and completion of leaf development, based on on‐the‐ground surveys conducted each spring since 1990.

At Howland Forest, the time series of *G*_cc_ and GRVI showed strong seasonality in canopy color, with both color indices following approximately sinusoidal patterns, reaching a minimum in winter and a maximum in summer (Fig. 2). On days with snow on the canopy (open symbols), *G*_cc_ was shifted downward by a relatively small amount relative to *G*_cc_ measured on days without snow on the canopy (blue symbols). By comparison, when there was snow on the canopy, GRVI was shifted upward to levels that were more similar to summer values. Filtering data for snow on canopy is therefore important for *G*_cc_ but critical for GRVI. Even when there was no snow on the canopy, GRVI appeared to be more variable, day‐to‐day, than *G*_cc_, with some obvious outliers. There was less year‐to‐year variation in the winter minimum of *G*_cc_ than GRVI, but overall the two time series were well correlated (*r* = 0.89).

The data indicate that the onset of increases in *G*_cc_ and GRVI occurred independently of the production of new foliage, as determined by on‐the‐ground phenological surveys (Fig. 2). Notably the date of budburst by the dominant conifer species, red spruce and eastern hemlock, occurs after the point when more than half of the annual seasonal amplitude of these indices has already been reached.

#### Harvard Forest

**Fig. 3:**
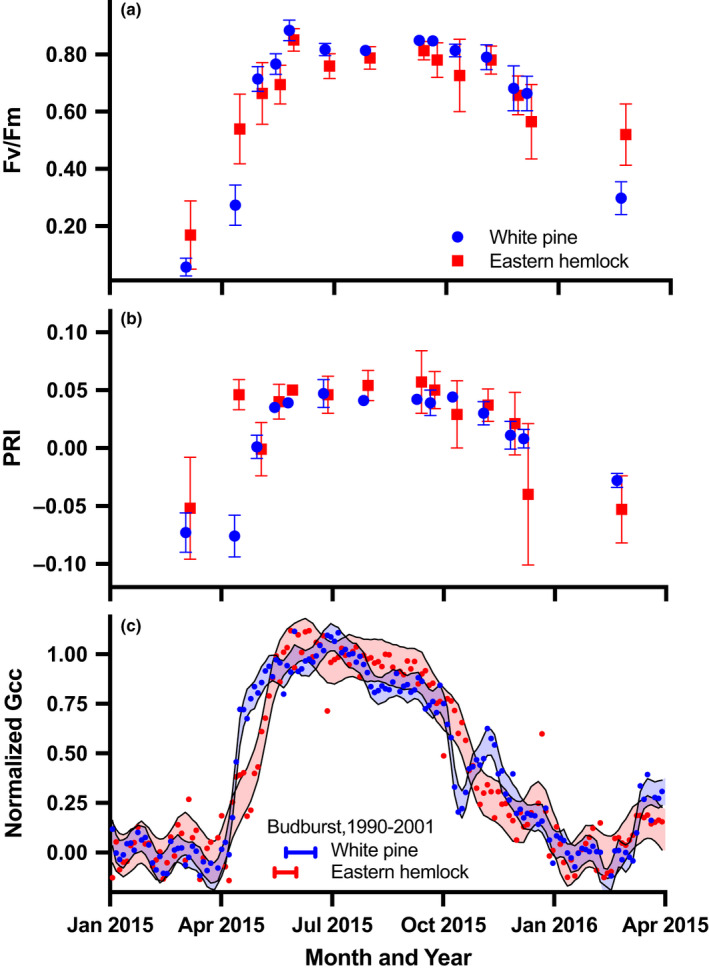
Seasonal variation in leaf‐level physiological measurements for white pine and eastern hemlock at Harvard Forest. (a) Chlorophyll fluorescence *F*_v_ */F*_m_; (b) the photochemical reflectance index, PRI; (c) the green chromatic coordinate (*G*_cc_), normalized by observed seasonal maxima and minima to fall within the range 0–1. In (c), the horizontal bars indicate the mean dates of budburst (production of new foliage) for each species, over the period 1990–2001 (ground observations were discontinued before the start of the present study). The errors bars in (a) and (b) show the SD of the observations.

At Harvard Forest, leaf‐level measurements on white pine and eastern hemlock showed strong seasonal patterns in two key physiological indices, chlorophyll fluorescence, *F*_v_ */F*_m_, and the PRI (Fig. 3). The value of *F*_v_ */F*_m_ indicates that leaf‐level changes in the quantum efficiency of photosystem II (PS II; Bolhàr‐Nordenkampf *et al*., 1989) occurred in parallel with changes in *G*_cc_ over the course of the year. PRI suggests that the changes in *G*_cc_ were associated with variation in the chl : car ratio (Garbulsky *et al*., 2011). As was the case at Howland Forest (Fig. 2), increases in *G*_cc_ (normalized in Fig. 3c to account for the differences in winter baseline and summer maxima between the two species) began many weeks before the production of new foliage occurred in June.

#### Niwot Ridge

**Fig. 4:**
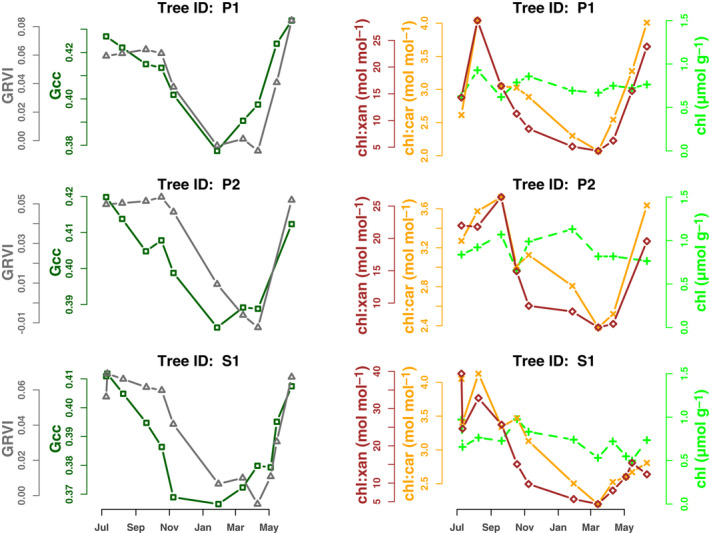
Seasonal patterns in phenocam‐derived canopy color indices (*G*_cc_ and GRVI), and pigment contents and ratios, for three trees (two lodgepole pine: P1 and P2, and one Engelmann spruce: S1) in the field of view of the niwot5 phenocam. For additional pigment content (total carotenoids and total xanthophylls) data, see Supporting Information Fig. S3.

At Niwot Ridge, our analysis of the temporal patterns in leaf pigments highlights the strong relationship between canopy color and leaf‐level pigment pools and ratios. First, the seasonal patterns in both color indices (*G*_cc_ and GRVI) and pigment data were highly consistent across the three trees (Fig. 4; two lodgepole pines: P1 and P2, and one Engelmann spruce: S1) in the field of view of the *niwot5* phenocam, with one exception: chlorophyll content did not vary across individuals, or over time. This is consistent with results presented by Bowling *et al*. (2018) using data from a previous year.

**Fig. 5:**
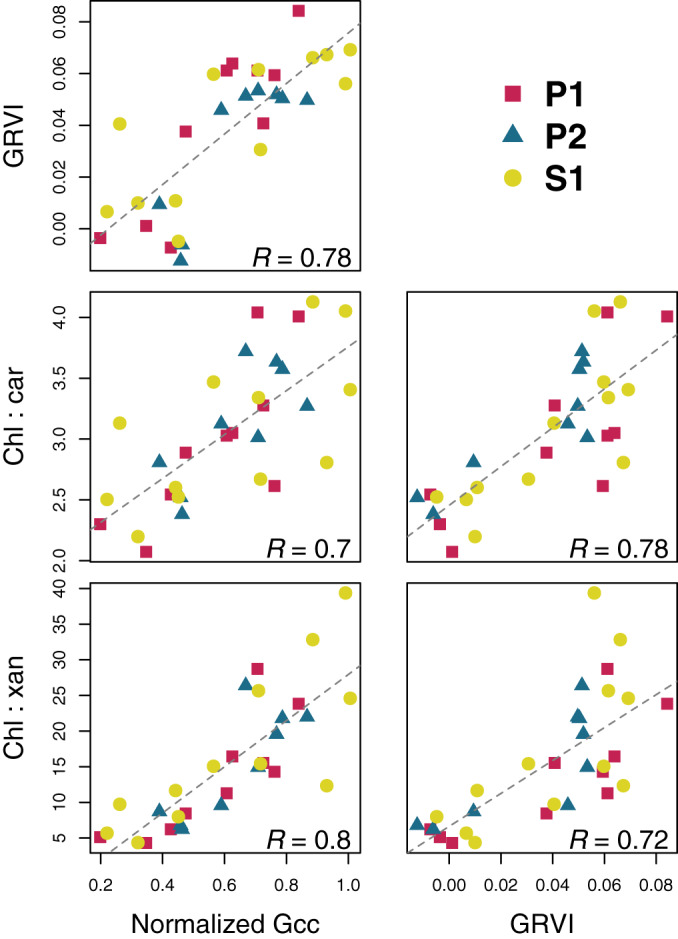
Correlation of canopy color indices and pigment ratio data for Niwot Ridge. Data are plotted using different symbols for each of the three trees (two lodgepole pine: P1 and P2, and one Engelmann spruce: S1) within the *niwot5* phenocam field of view. Rather than presenting the full correlation matrix (see Supporting Information Fig. S3), here we show only the strongest correlations. To account for the different seasonal maxima and minima of green chromatic coordinate (*G*_cc_) across different trees, the values were normalized.

Second, the seasonal patterns of variation in *G*_cc_ correlated best with those for the chl : xan ratio, while GRVI correlated best with the chl : car ratio (Fig. 5). These results show clear covariation on seasonal timescales between leaf‐level pigments and canopy color as quantified from phenocam imagery.

**Fig. 6:**
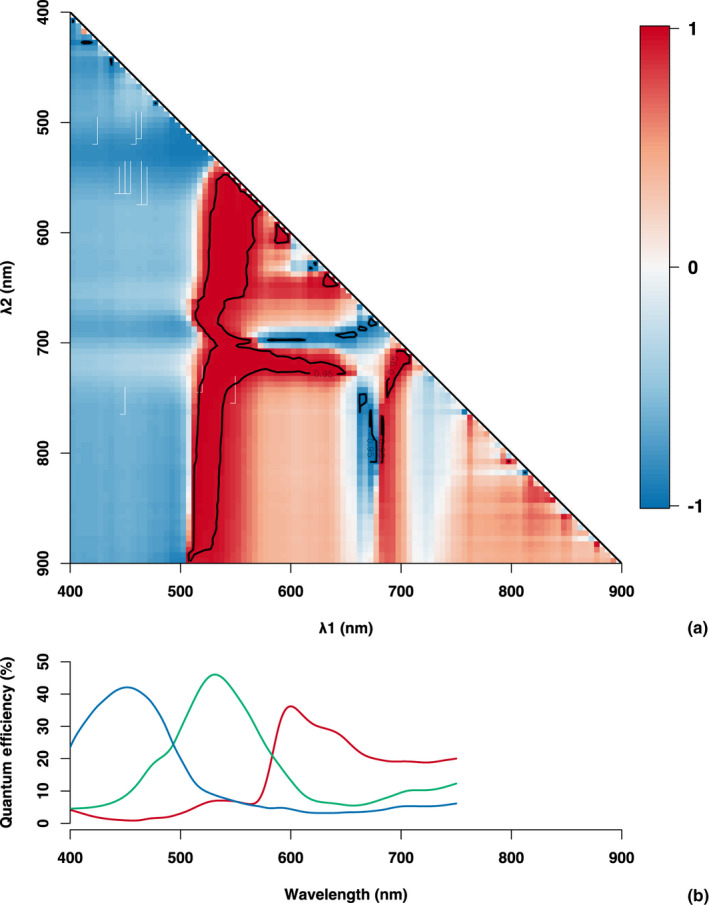
(a) Heatmap plot showing Pearson (*r*) correlation of normalized difference indices (Eqn 3), *λ*_1_ and *λ*_2_ wavelengths, with leaf‐level measurements of chl : car pigment ratio conducted over the course of the year. The white contour line indicates |*r*| = 0.95. (b) Quantum efficiency of PhenoCam digital cameras for each color channel. The hyperspectral reflectance data were obtained from a 2D scanning telescope, PhotoSpec.

We complement the earlier analysis of broad‐band changes in canopy color with a more detailed analysis using hyperspectral vegetation reflectance data (Fig. 6). Across all wavelength combinations, we found *λ*_1_ ~ 530 nm (green) and *λ*_2_ ~ 690 nm (red) resulted in high correlation (*r* > 0.95) between a two‐wavelength NDI and the chl : car ratio, highlighting the importance of seasonal changes in leaf pigment content as a likely mechanism driving changes in spectral reflectance, and the potential to detect these changes with color‐based indices (specifically GRVI, given the optimal bands for *λ*_1_ and *λ*_2_) retrieved from camera imagery. There were a number of narrowly‐defined spectral ranges (e.g. for any *λ*_2_, 500 < *λ*_1_ < 550 nm and 675 < *λ*_1_ < 700 nm, and also 700 < *λ*_2_ < 725 nm when 525 < *λ*_1_ < 650) where correlations with chl : car were high. We further note that for any value of *λ*_2_ when *λ*_1_ < 500 nm, correlation of NDI with chl : car was not as strong as with the green–red band combinations.

### Links between seasonal changes in canopy color and photosynthetic capacity

**Fig. 7:**
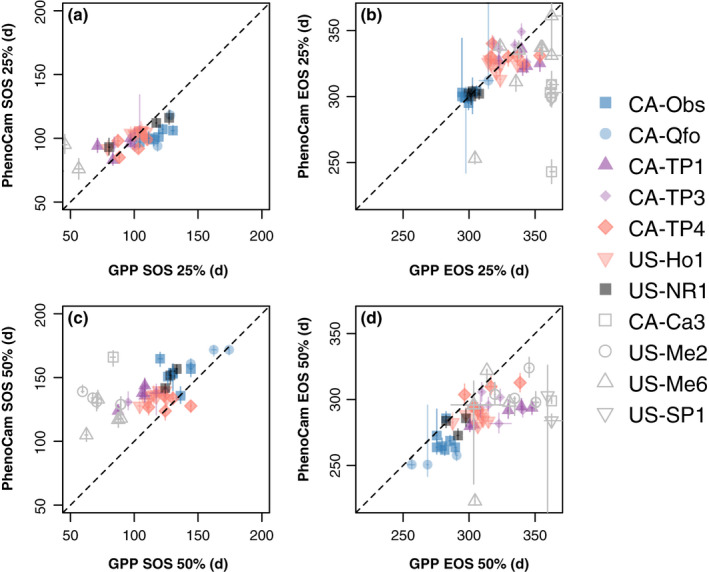
Start‐of‐season (SOS) and end‐of‐season (EOS) derived from PhenoCam reflectance indices and from gross primary productivity (GPP) were highly correlated. Vertical axes show PhenoCam‐based transition dates. Horizontal axes show GPP‐based transition dates. Upper panels (a, b) are based on the 90^th^ percentile of the green chromatic coordinate (*G*_cc_) time series. Lower panels (c, d) are based on the mean green–red vegetation index (GRVI) time series. Individual years for each site are shown separately. Uncertainties are shown as error bars. Warm sites, where photosynthesis occurs year‐round, are shown with hollow symbols.

SOS and EOS transition dates derived from *G*_cc_ and GRVI were generally aligned with the corresponding dates indicating the start and end of photosynthetic activity, as estimated from GPP (Fig. 7). But there were obvious outliers to these relationships at sites which were not fully winter dormant; although photosynthetic capacity does decline in winter at the warm sites, it does not shut down completely, and thus the concept of SOS and EOS dates is not applicable (even though seasonal variation in canopy color was observed even at the warmest site, US‐SP1). Our analysis is therefore restricted to the remaining seven winter‐dormant sites, which ranged across 14° in latitude, 37° in longitude, and more than 8°C in MAT. SOS and EOS transition dates varied by more than 2 months across site‐years (*n* = 38 and *n* = 34, respectively).

As described in the Methods section, we found that many different calculations could provide PhenoCam‐based transition dates that were well‐correlated with GPP‐based transition dates, although some methods were superior (Table S3). The best methods resulted in transition dates for both *G*_cc_ and GRVI that were well‐correlated with GPP‐based dates for both SOS (*r* = 0.70 and *r* = 0.69, respectively) and EOS (*r* = 0.73 and *r* = 0.74) (Fig. 7). However, the root‐mean‐square error (RMSE) for *G*_cc_ SOS (6 d) was substantially smaller than for *G*_cc_ EOS (11 d), and for either GRVI SOS (10 d) or EOS (11 d).

### Temperature‐based modeling of evergreen phenology

**Fig. 8:**
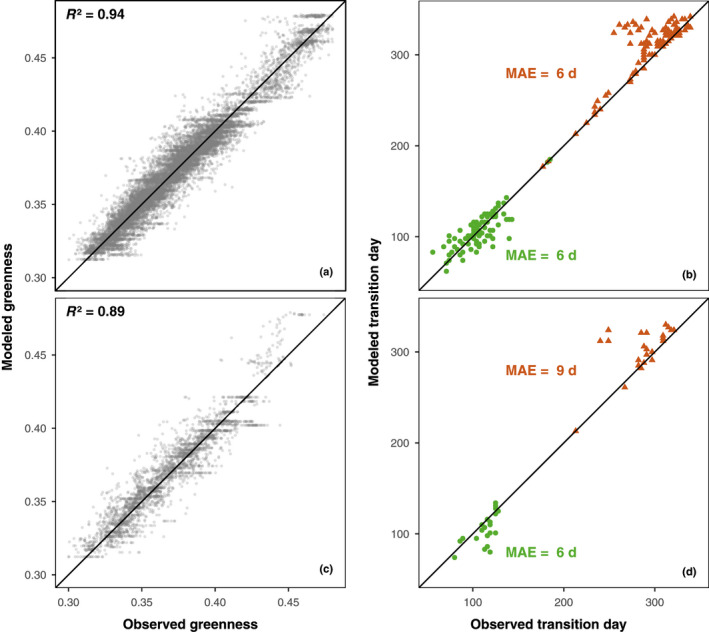
Validations of the phenology model for (a, b) in‐sample and (c, d) out‐of‐sample data. The plots on the left (a, c) are modeled vs observed greenness values. The plots on the right (b, d) are modeled vs observed transition dates values. MAE stands for median absolute error. Green circles and orange triangles on the right‐hand‐side panels indicate spring and autumn transition dates, respectively. Horizontal features in (a) and (c) are due to the minimum and maximum greenness values in the model parameters.

Our temperature‐based phenology model performed well, in that it was able to reproduce both the seasonal trajectory of canopy color and transition dates for in‐sample data, as well as out‐of‐sample data (Fig. 8).

Despite the large climatological range, and differences in species composition across sites, the model consistently replicated the seasonal patterns of *G*_cc_ and successfully predicted the year held out for cross‐validation at each site. For example, US‐Mpj is a semi‐arid pinyon‐juniper forest in New Mexico, USA while CA‐Obs is a boreal spruce forest in Saskatchewan, Canada. The two contrasting sites exhibited distinct trends in their seasonal change of canopy color (Fig. S2). The seasonal amplitude of greenness at CA‐Obs was more than twice as much as that at US‐Mpj, and green‐up and green‐down occurred at a faster rate at CA‐Obs than at US‐Mpj. The model performed almost equally well at both sites (Fig. S2).

Our temperature‐based model closely tracked the timing and the rate of changes in greenness for all site‐years. The values of *R* ^2^ for calibration data (82% of the data) and validation data (18% of the data) were 0.94 and 0.89, respectively (Fig. 8). The spring and autumn transition dates extracted from the modeled time series also matched well with the transition dates derived from the *G*_cc_ time series. The median absolute error was 6 d for both spring and autumn transition dates when we used the calibration data. For the validation data, the median absolute errors were 6 and 9 d for spring and autumn transition dates, respectively. Model parameters were generally similar across different species and climate regimes (see Table S4). More specifically, *G*_min_, *G*_max_ and *D* showed small variability across sites. The spring threshold temperature (*θ*_1_) expressed higher variability than the autumn threshold temperature (*θ*_2_), but the variability of temperature sensitivity coefficient of spring (*ρ*_1_) was larger than that of autumn (*ρ*_2_). Overall, the model parameters did not vary in relation to climate (MAT or MAP) or site location (latitude, longitude, elevation) factors, but as the PhenoCam data archive grows, it may be possible to identify biogeographic patterns in model parameters.

## Discussion

Our study shows that changes in evergreen conifer canopy color that occur on seasonal timescales are associated with variation in leaf pigment ratios. Furthermore, our results show that changes in canopy color can be detected from digital camera imagery, and SOS and EOS transition dates derived from canopy color metrics align with corresponding dates representing the start and end of photosynthetic activity in winter‐dormant ecosystems (Gamon *et al*., 2016; Wong *et al*., 2020). Finally, the seasonal trajectory of changes in canopy color can be simulated with a temperature‐based model which likewise successfully reproduces the timing of SOS and EOS transition dates across a wide range of evergreen ecosystems. Our work builds on previous studies (Richardson *et al*., 2009a; Toomey *et al*., 2015; Bowling *et al*., 2018; Liu *et al*., 2020) by providing new insight into the underlying mechanisms and drivers of the observed relationship between canopy color and photosynthesis, and highlighting the broader generality of these results across North America.

### The seasonality of canopy color variation is directly linked to photochemical processes

We found that two visible‐wavelength spectral indices, *G*_cc_ and GRVI, reveal strong seasonal variation in color of evergreen conifer canopies. This variation occurred independently of new leaf development in spring (Figs 2, 3c; see also Zhang *et al*., 2020). Seasonal variation in canopy color occurred in tandem with shifts in the quantum efficiency of PS II photochemistry as measured by *F*_v_ */F*_m_ (Fig. 3a), as well as PRI (Fig. 3b) and leaf pigment pools and their ratios (Fig. 5). The leaf‐level processes associated with seasonal upregulation and downregulation of the photosynthetic machinery, and related mechanisms of photoprotection, in evergreen conifer forests have been well‐documented (Busch *et al*., 2009; Fréchette *et al*., 2015, 2016; Wong *et al*., 2019; Grebe *et al*., 2020). Downregulation of photosynthesis involves inactivation of PS II reaction centers and re‐organization of light harvesting complexes to function for energy dissipation (Ensminger *et al*., 2006). Ensminger *et al*. (2004) reported that the springtime increase in NEE of a Scots pine forest, which occurred as temperatures rose above 0°C, is correlated with detectable changes in leaf pigments, *F*_v_ */F*_m_, and chloroplast protein synthesis. This previous work provides context for the results and support for the conclusions drawn here.

The PRI has been shown to be a reliable index of photosynthetic radiation use efficiency in evergreen forests (Garbulsky *et al*., 2011; Soudani *et al*., 2014). Although originally conceived to detect changes in xanthophyll cycle epoxidation state occurring on minutes to diurnal timescales (Gamon *et al*., 1992), on seasonal timescales PRI is more indicative of shifts in the chl : car ratio that occur independently of the xanthophyll cycle (Wong & Gamon, 2015a). Upregulation and downregulation of photosynthesis occurs as a complex process involving a number of discrete components that are coordinated but operate on different timescales (Wong & Gamon, 2015b; see also Busch *et al*., 2009; Fréchette *et al*., 2016). Nevertheless, on seasonal timescales PRI and CCI are reliable indicators of potential photosynthetic activity because of their sensitivity to the chl : car ratio (Wong & Gamon, 2015b; Gamon *et al*., 2016; Wong *et al*., 2020). Likewise, the indices used here to track canopy color, *G*_cc_ and GRVI, mimic the seasonality of PRI and *F*_v_ */F*_m_ (Fig. 3) and are correlated with the chl : xan and chl : car ratios (Fig. 5), thereby establishing a plausible link between canopy color and photochemical processes.

### Changes in pigment pools and ratios are detected from canopy‐scale near‐surface remote sensing

Near‐surface remote sensing at the canopy level presents new challenges that are not typically an issue with leaf‐level spectral measurements. These include the effects of canopy structure and nonphotosynthetic biomass, as well as changing illumination geometry and weather. Our study builds on previous work that has linked leaf‐ and canopy‐level spectral measurements to seasonal co‐variation in pigments and photosynthesis (Wong *et al*., 2019, 2020). In addition to showing that vegetation indices calculated from hyperspectral PhotoSpec data correlated well with pigment pools and ratios, we found that color indices derived from broadband phenocam imagery performed almost equally well (Fig. 5 **)**. Phenocam indices have been shown to have a nonlinear relationship with pigment concentrations in senescing deciduous foliage (Junker & Ensminger, 2016; Liu *et al*., 2018). Thus, changes in pigment pools and ratios are associated with changes in foliage color that can be detected using broad‐band visible‐wavelength color indices.

Snow is problematic for remote sensing of evergreen forests (Jönsson *et al*., 2010); it obscures the vegetation of interest and confounds the spectral signature. This is particularly an issue with GRVI (Fig. 3). An advantage of the phenocam approach is that images can be visually inspected to identify days when there is snow on the canopy, and the affected images removed from the analysis (Richardson *et al*., 2018b). Automated methods have also been developed to identify snowy images (Kosmala *et al*., 2018). By comparison, snow cover data products from satellite remote sensing (e.g. MODIS MOD10A1 and MYD10A1) may fail to detect discontinuous snow cover (Kosmala *et al*., 2018), which could still adversely impact spectral indices. Future studies that attempt to link data from orbiting sensors to tower‐based carbon and water flux measurements could benefit from making use of the imagery from collocated phenocams to identify snowy days.

### Canopy color indices derived from digital camera imaging can be used to characterize photosynthetic phenology

Although the term ‘phenology’ is typically applied to visually observable developmental events, such as budburst and flowering, it has also been used to describe the seasonality of physiological processes at leaf‐to‐ecosystem scales, e.g. ‘photosynthetic phenology’ (Gu *et al*., 2009; Richardson *et al*., 2013a; Gamon *et al*., 2016). The timing of the start and end of the photosynthetically active season plays a large role in controlling annual ecosystem productivity, and how productivity varies in time and space (Richardson *et al*., 2010a). Low‐cost sensors that could be used to characterize photosynthetic phenology would be of value for scaling photosynthetic uptake from a small set of intensively‐monitored sites with eddy covariance instrumentation to a much larger, more spatially extensive observation network.

Previous studies have highlighted the ability of CCI and PRI, measured at both the leaf‐ (Eitel *et al*., 2019; Wong *et al*., 2019) and canopy‐level (Gamon *et al*., 2016), to provide robust measures of the SOS and EOS dates for photosynthetic phenology. The results presented here show that *G*_cc_ and GRVI offer similar potential. Our analysis includes sites across a wide geographic range in North America, with SOS and EOS dates both varying by up to 70 d across sites and years (Fig. 7). We acknowledge that relationships between our phenocam indices and GPP‐derived transition dates broke down at warm sites where photosynthesis was maintained year‐round (albeit at a reduced rate in winter), even though *G*_cc_ and GRVI indicated variation in canopy color. One hypothesis to explain this pattern is that the photoprotective mechanisms at these warmer sites are not upregulated (or downregulated) to the same degree that they are in colder sites, and this drives differences in the relationship between reflectance characteristics and photochemistry. Nevertheless, our results support the idea that phenocams are low‐cost but sensitive instruments that have a wide applicability in environmental monitoring (Richardson, 2019). Here, we have specifically shown the potential value of phenocams for identifying seasonal patterns in evergreen conifer forests.

### A temperature‐based model can simulate the trajectory of seasonal changes in canopy color

We presented a dynamic modeling framework for simulating canopy color, and by proxy photosynthetic activity, that can be flexibly adapted to larger scales and applications. The structure of our model has roots in previous representations of evergreen phenology (Hänninen & Kramer, 2007; Hänninen, 2016). The strong sensitivity of increases in canopy greenness to warm temperature in spring, and decreases in canopy greenness in autumn that are driven by cold temperature, is consistent with our understanding of the role of temperature and photoperiod in driving evergreen seasonality (Tanja *et al*., 2003; Ensminger *et al*., 2004; Fréchette *et al*., 2016). The results are also consistent with previous empirical analyses of phenocam data, which found that interannual variation in evergreen conifer SOS and EOS dates was mostly explained by air temperature during a narrow window preceding each transition (Richardson *et al*., 2019). But, similar to Tanja *et al*. (2003), we found that it was necessary to parameterize our model separately at each site. And, although we had anticipated that unusually cold temperature in spring (or warm temperatures in autumn) might cause temporary reversal of the overall upward (downward) trajectory, we were unable to identify the associated temperature thresholds, perhaps because at the site level, such events are relatively rare. While further work is therefore needed to develop an improved model that generalizes well across sites, this framework may ultimately have application in global land surface models in which the seasonality of evergreen conifer forests is poorly represented (Lawrence *et al*., 2011; Richardson *et al*., 2012; Peaucelle *et al*., 2019) and in incorporating potential effects from other factors such as daylength (Way & Montgomery, 2015).

### Conclusion

The novel contribution of our study is the finding that seasonal pigment changes in evergreen conifers are detectable at the canopy level using imagery from low‐cost, commercially available digital cameras. Given the low cost of digital cameras, our work highlights the potential value in co‐locating phenocams with more advanced instruments such as solar induced fluorescence (SIF). SIF has been shown to track changes in canopy‐level photosynthetic capacity across a range of timescales in evergreen ecosystems (Zuromski *et al*., 2018; Magney *et al*., 2019). We note that many leaf‐level studies have found it advantageous to simultaneously measure both *F*_v_ */F*_m_ and PRI to fully understand the factors that are regulating photosynthesis at different times of the year (Busch *et al*., 2009; Fréchette *et al*., 2016; Wong *et al*., 2019). Likewise, installing phenocams at sites where SIF instrumentation is already installed may have great value for offsetting the specific limitations of each method. These measurements would be complementary and not redundant and would allow independent quantification of the temporal kinetics of both chlorophyll fluorescence and leaf pigmentation.

## Author contributions

ADR and DRB planned the IMGG workshop and designed the research. BS, DRB, RC, BAL, TSM, CF, JCY, AMY and KH conducted research. MAA, TAB, PDB, RB, RJ, DYH, BEL and ZN contributed tower measurements. BS, RC, DRB and ADR analyzed data. BS, DRB and ADR drafted the manuscript with input from RC, BAL, TSM and KH. All authors provided feedback on manuscript drafts and approved the manuscript for submission.

## Supporting information


**Fig. S1** Illustration of workflow for processing tower‐measured fluxes of net ecosystem exchange (NEE) of CO_2_ to extract seasonality of photosynthetic capacity and associated transition dates.
**Fig. S2** Temperature‐based phenology model captures the seasonal trajectory of changes in canopy color for two sites with strong climatological and species composition differences.
**Fig. S3** Seasonal patterns in phenocam‐derived canopy color indices (*G*_cc_ and GRVI), and pigment contents and ratios, for three trees (two lodgepole pine: P1 and P2, and one Engelmann spruce: S1) in the field of view of the niwot5 phenocam.
**Fig. S4** Heatmaps show correlation values between color‐ and pigment‐based indices.
**Table S1** Metadata for eddy covariance study sites.
**Table S2** Metadata for PhenoCam study sites.
**Table S3** Evaluation of correlation of start‐of‐season (SOS) and end‐of‐season (EOS) transition dates, derived from PhenoCam imagery, with corresponding dates derived from tower‐based estimates of gross primary production (GPP).
**Table S4** List of the fitted model parameters.
Please note: Wiley Blackwell are not responsible for the content or functionality of any Supporting Information supplied by the authors. Any queries (other than missing material) should be directed to the *New Phytologist* Central Office.

## Data Availability

Freely available code and data can be accessed from https://github.com/bnasr/ENPhenology.
