# Supplementary material for: Seasonal variation in the canopy color of temperate evergreen conifer forests
Source: New Phytol. 2020 Dec 1;229(5):2586–600. doi: 10.1111/nph.17046 (PMC7898516; doi:10.1111/nph.17046)
Supplement: Supplementary file 1 — Fig. S1 Illustration of workflow for processing tower‐measured fluxes of net ecosystem exchange (NEE) of CO2 to extract seasonality of photosynthetic capacity and associated transition dates. Fig. S2 Temperature‐based phenology model captures the seasonal trajectory of changes in canopy color for two sites with strong climatological and species composition differences. Fig. S3 Seasonal patterns in phenocam‐derived canopy color indices (G cc and GRVI), and pigment contents and ratios, for three trees (two lodgepole pine: P1 and P2, and one Engelmann spruce: S1) in the field of view of the niwot5 phenocam. Fig. S4 Heatmaps show correlation values between color‐ and pigment‐based indices. Table S1 Metadata for eddy covariance study sites. Table S2 Metadata for PhenoCam study sites. Table S3 Evaluation of correlation of start‐of‐season (SOS) and end‐of‐season (EOS) transition dates, derived from PhenoCam imagery, with corresponding dates derived from tower‐based estimates of gross primary production (GPP). Table S4 List of the fitted model parameters. Please note: Wiley Blackwell are not responsible for the content or functionality of any Supporting Information supplied by the authors. Any queries (other than missing material) should be directed to the New Phytologist Central Office. [file NPH-229-2586-s001.pdf]

New Phytologist Supporting Information

Article title: **Seasonal variation in the canopy color of temperate evergreen conifer forests**

Authors: Bijan Seyednasrollah, David R. Bowling, Rui Cheng, Barry A. Logan, Troy S. Magney, Christian Frankenberg, Julia C. Yang, Adam M. Young, Koen Hufkens, M. Altaf Arain, T. Andrew Black, Peter D. Blanken, Rosvel Bracho, Rachhpal Jassal, David Y. Hollinger, Beverly E. Law, Zoran Nesic, and Andrew D. Richardson

Article acceptance date: 13 October 2020

The following Supporting Information is available for this article:

Figure S1 Illustration of workflow for processing tower-measured fluxes of net ecosystem exchange (NEE) of CO_2 to extract seasonality of photosynthetic capacity and associated transition dates.

Figure S2 Temperature-based phenology model captures the seasonal trajectory of changes in canopy color for two sites with strong climatological and species composition differences.

Figure S3 Seasonal patterns in phenocam-derived canopy color indices (G_{cc} and GRVI), and pigment contents for three trees (two lodgepole pine: P1 and P2, and one Engelmann spruce: S1) in the field of view of the *niwot5* phenocam.

Figure S4 Heatmaps show correlation values between color- and pigment- based indices.

Table S1 Metadata for eddy covariance study sites

Table S2 Metadata for PhenoCam study sites

Table S3 Evaluation of correlation of start-of-season (SOS) and end-of-season (EOS) transition dates, derived from PhenoCam imagery, with corresponding dates derived from tower-based estimates of gross primary productivity (GPP).

Table S4 List of the fitted model parameters.

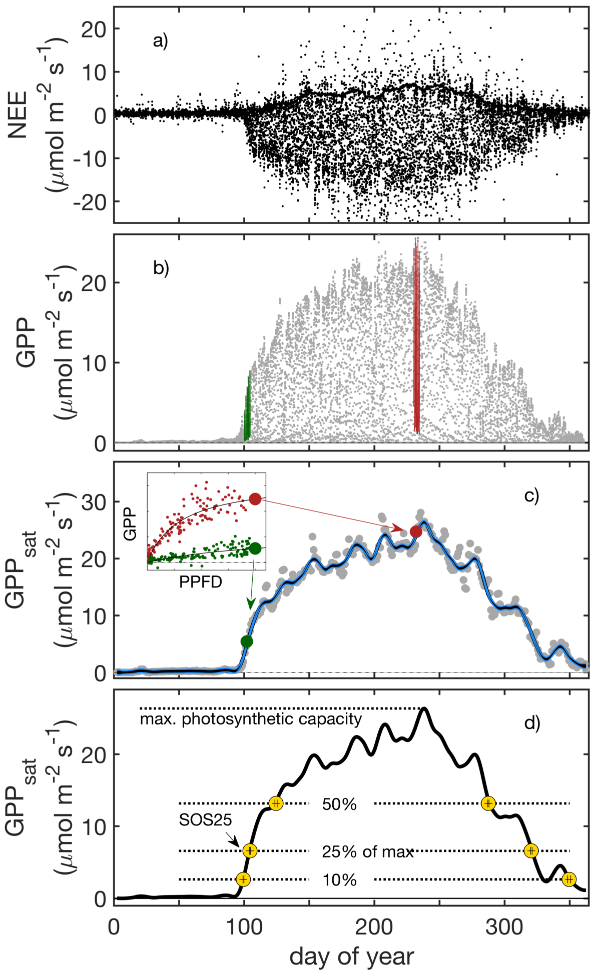

Figure S1 Illustration of workflow for processing tower-measured fluxes of net ecosystem exchange (NEE) of CO_2 to extract seasonality of photosynthetic capacity and associated transition dates. The example is based on data from Niwot Ridge. (a) 30-minute time series of NEE of CO_2, after u* filtering to remove nighttime data recorded under conditions of low turbulence; (b) 30-minute time series of gross primary productivity (GPP), calculated as the difference between estimated ecosystem respiration and measured NEE; (c) estimation of seasonal trajectory of canopy-level photosynthetic capacity index (GPPsat) from analysis of light response curves (inset with colors indicating time period); (d) determination of start of season (SOS) and end of season (EOS) transition dates from the time series of GPPsat.

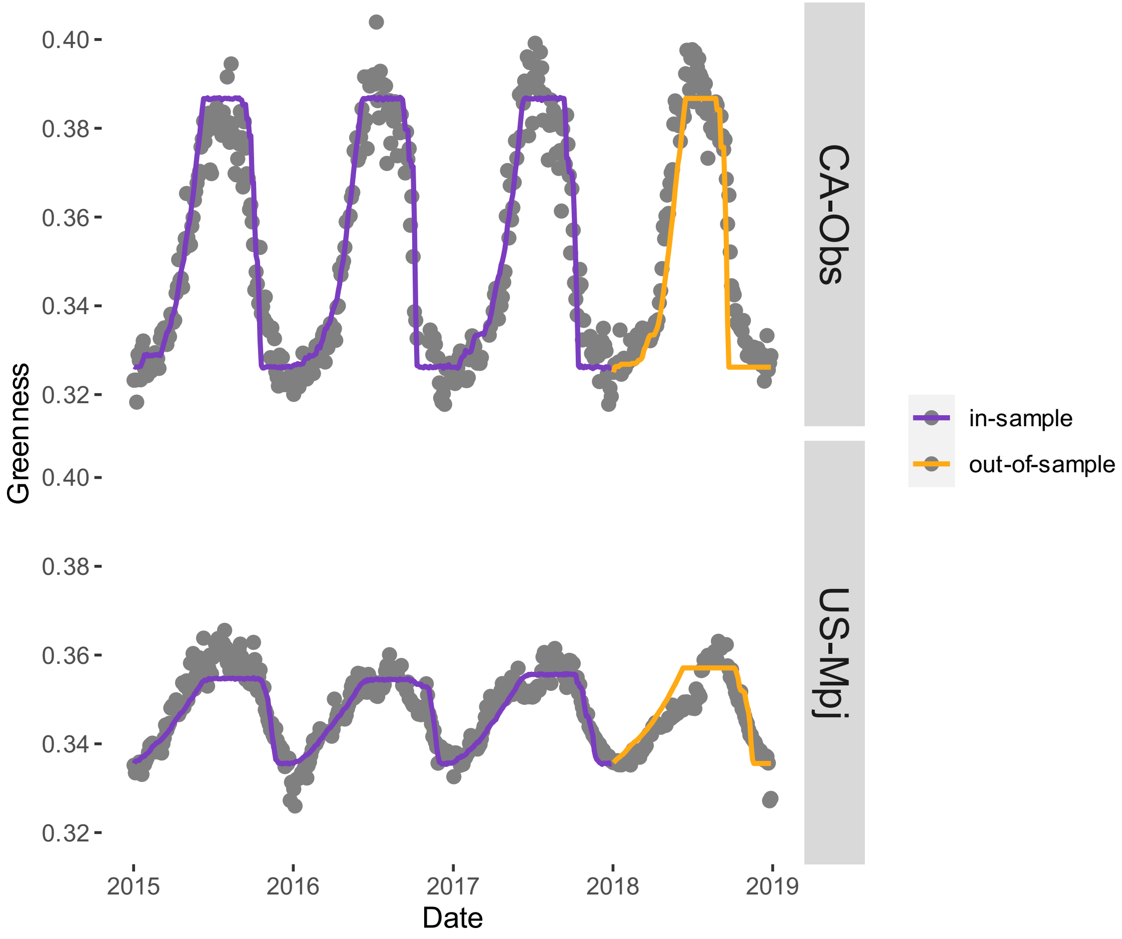

Figure S2 Temperature-based phenology model captures the seasonal trajectory of changes in canopy color for two sites with strong climatological and species composition differences.

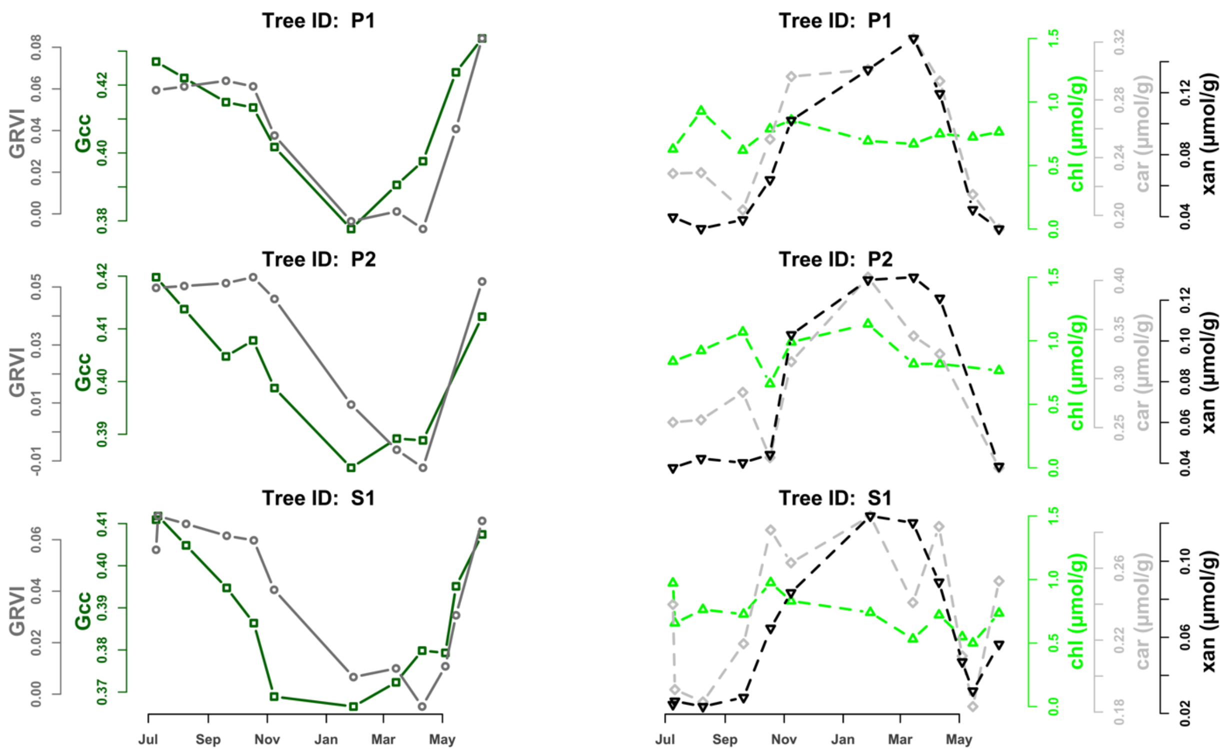

Figure S3 Seasonal patterns in phenocam-derived canopy color indices (G_{cc} and GRVI), and pigment contents for three trees (two lodgepole pine: P1 and P2, and one Engelmann spruce: S1) in the field of view of the *niwot5* phenocam.

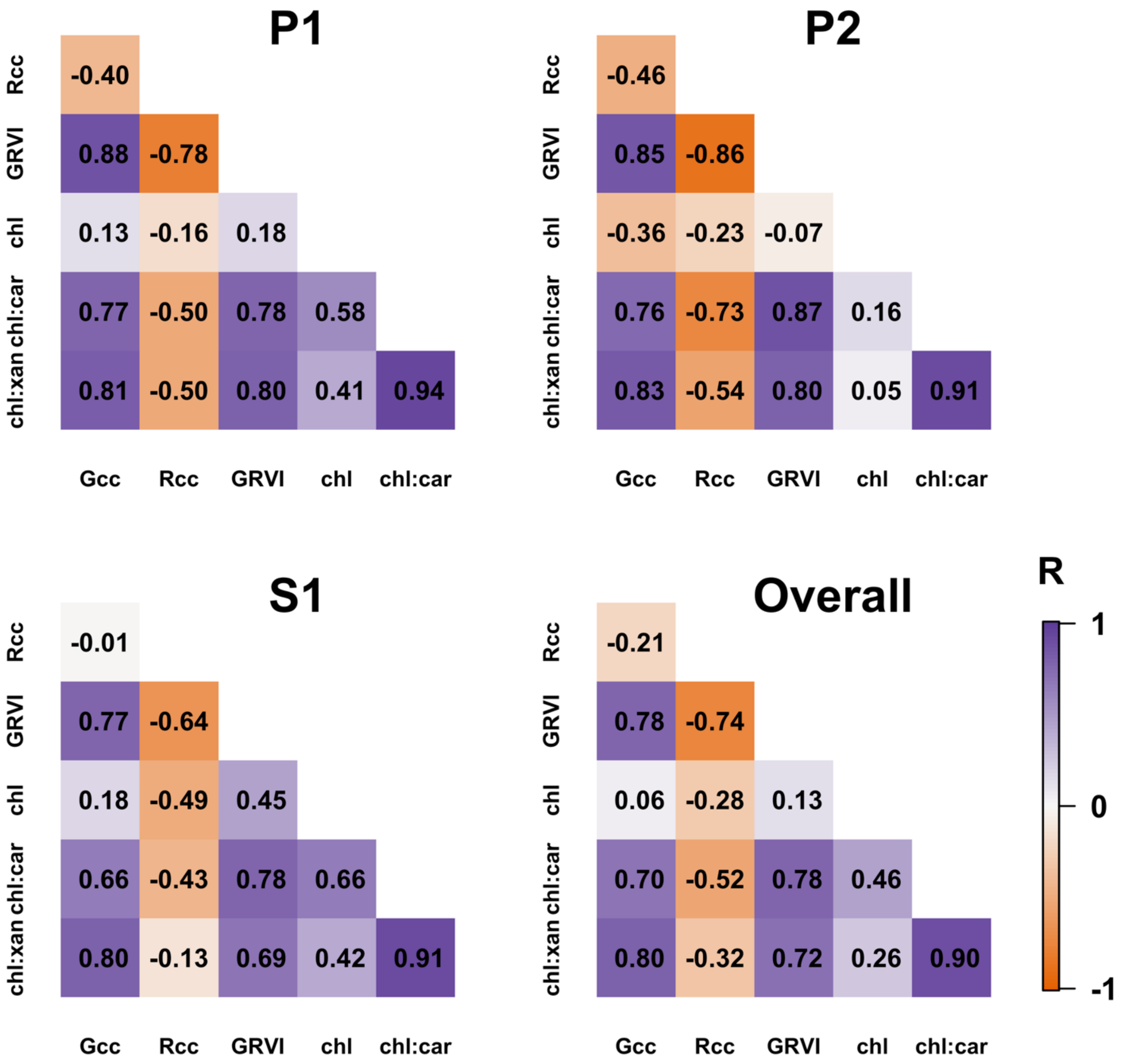

Figure S4 Heatmaps show correlation values between color- and pigment- based indices.

Table S1 Metadata for eddy covariance study sites

Description /	Citations	Site Code		Lat. (°)	Lon. (°)	Alt. (m)	Mean Temperature (°C)			MAP (mm)	Snow Days	Dominant Spices
		FLUXNET	PhenoCam				Annual	January	July			
Pole sapling Douglas-fir stand, British Columbia[1]	Jassal *et al.* (2009)	CA-Ca3	vancouverisland	49.53	-124.90	173	9.9	2.4	17.4	1676	8	*Pseudotsuga menziesii*
Western Boreal, Mature Black Spruce, Saskatchewan[2]	Liu *et al.* (2019)	CA-Obs	canadaOBS	53.98	-105.11	628	0.8	-17.8	16.7	405	115	*Picea mariana, Larix laricina*
Eastern Boreal, Mature Black Spruce, Quebec[3]	Margolis (2003) Bergeron *et al.* (2007)	CA-Qfo	chibougamau	49.69	-74.34	382	-0.4	-19.3	16.1	962	117	*Picea mariana*
Turkey Point 2002 Plantation, Ontario[4]	Arain (2003a) Peichl *et al.* (2010) Chan *et al.* (2018)	CA-TP1	turkeypointenf02	42.66	-80.55	265	8	-4.2	21.4	1036	30	*Pinus strobus*
Turkey Point 1974 Plantation, Ontario[5]	Arain (2003b) Peichl *et al.* (2010)	CA-TP3	turkeypointenf74	42.70	-80.34	216	8	-4.4	21.3	1036	18	*Pinus strobus*
Turkey Point 1939 Plantation, Ontario[6]	Arain (2003c) Arain & Restrepo-Coupe (2005)	CA-TP4	turkeypointenf39	42.71	-80.35	232	8	-4.4	21.2	1036	34	*Pinus strobus, Abies balsamea, Quercus velutina, Quercus alba, Acer rubrum*
Howland Forest, Maine[7]	Hollinger (1996) Hollinger *et al.* (1999) Richardson *et al.* (2019a)	US-Ho1	howland1	45.20	-68.74	60	5.3	-9.2	19.8	1070	51	*Picea rubens, Tsuga canadensis, Acer rubrum*
Metolius mature ponderosa pine, Oregon[8]	Law (2002) Law & Berner (2015) Kwon *et al.* (2018)	US-Me2	oregonMP	44.45	-121.55	1253	6.3	-0.5	16.7	523	49	*Pinus ponderosa*
Metolius Young Pine Burn, Oregon[9]	Law (2010) Law & Berner (2015) Ruehr *et al.* (2012)	US-Me6	oregonYP	44.32	-121.60	998	7.6	0.4	17.7	494	45	*Pinus ponderosa*
Niwot Ridge Forest, Colorado[10]	Blanken *et al.* (1998) Burns *et al.* (2015)	US-NR1	niwot5	40.03	-105.54	3050	1.5	-6.8	12.7	800	71	*Picea engelmannii, Abies lasiocarpa, Pinus contorta*
Austin Cary, Slashpine[11]	Martin (2000)	US-SP1	Austincary	29.73	-82.21	50	20.1	12.5	27.4	1310	4	*Pinus palustris, Pinus elliotti*

1. https://doi.org/10.17190/AMF/1480302
2. https://doi.org/10.17190/AMF/1375198
3. https://doi.org/10.17190/AMF/1246829
4. https://doi.org/10.17190/AMF/1246009
5. https://doi.org/10.17190/AMF/1246011
6. https://doi.org/10.17190/AMF/1246012
7. https://doi.org/10.17190/AMF/1246061
8. https://doi.org/10.17190/AMF/1246076
9. https://doi.org/10.17190/AMF/1246128
10. https://doi.org/10.17190/AMF/1246088
11. https://doi.org/10.17190/AMF/1246100

Table 2 Metadata for PhenoCam study sites

Site Code		Lat (°)	Lon (°)	Alt. (m)	MAT (°C)	MAP (mm)	Acknowledgements
PhenoCam	FLUXNET						
canadaOBS	CA-Obs	53.98	-105.11	628	0.4	553	BERMS sites are funded through the Global Institute for Water Security (GIWS) at the University of Saskatchewan
harvardbarn	-	42.53	-72.18	350	7	1385	Research at Harvard Forest is partially supported through the National Science Foundation's LTER program (DEB-1237491).
harvardbarn2	-	42.53	-72.18	350	7	1385	Research at Harvard Forest is partially supported through the National Science Foundation's LTER program (DEB-1237491).
harvardhemlock	US-Ha2	42.53	-72.18	355	7	1385	Research at Harvard Forest is partially supported through the National Science Foundation's LTER program (DEB-1237491), and Dept. of Energy Office of Science (BER)
harvardhemlock2	US-Ha2	42.53	-72.17	355	7	1385	Research at Harvard Forest is partially supported through the National Science Foundation's LTER program (DEB-1237491).
howland1	US-Ho1	45.20	-68.74	80	6.1	1143	Research at Howland Forest is supported by the Office of Science (BER), US Department of Energy, and the USDA Forest Service's Northern Research Station.
huyckpreserveny	-	42.52	-74.15	478	6.85	1141	Research at the preserve is supported by the NSF Award #145544: Collaborative Research: IDBR: TYPE A. The NANAPHID: A novel aphid-like nanosensor network for real-time measurements of carbohydrates in live plant tissue, and the NSF MRI Award #72205: Acquisition of a Small Unmanned Aircraft System of Natural and Urban Ecosystem Studies and Risk Disaster Management
laclaflamme	-	47.32	-71.12	784	-0.1	1576	-
laurentides	-	45.98	-74.00	350	4.05	1222	-
missouriozarks	US-MOz	38.74	-92.20	219	12.75	1102	Research at the MOFLUX site is supported by the U.S. Department of Energy, Office of Science, Office of Biological and Environmental Research Program, Climate and Environmental Sciences Division through Oak Ridge National Laboratory's Terrestrial Ecosystem Science – Science Focus Area. ORNL is managed by UT-Battelle, LLC, for the U.S. Department of Energy under contract DE-AC05-00OR22725.
niwot2	US-NR1	40.03	-105.54	3050	1.8	863	The US-NR1 AmeriFlux site is currently supported by the U.S. DOE, Office of Science through the AmeriFlux Management Project (AMP) at Lawrence Berkeley National Laboratory under Award Number 7094866.
niwot3	US-NR1	40.03	-105.54	3050	1.8	863	The US-NR1 AmeriFlux site is currently supported by the U.S. DOE, Office of Science through the AmeriFlux Management Project (AMP) at Lawrence Berkeley National Laboratory under Award Number 7094866.
niwot5	US-NR1	40.03	-105.54	2993	1.8	863	The US-NR1 AmeriFlux site is currently supported by the U.S. DOE, Office of Science through the AmeriFlux Management Project (AMP) at Lawrence Berkeley National Laboratory under Award Number 7094866.

oregonMP	US-Me2	44.45	-121.55	1253	6.9	1158	Support for US-Me2 is provided from the Metolius Core Site Cluster by the DOE Office of Science Ameriflux Network Management Project
oregonYP	US-Me6	44.32	-121.6	977	8	893	Support for US-Me6 is provided from the Metolius Core Site Cluster by the DOE Office of Science Ameriflux Network Management Project
spruceA0EMI	-	47.50	-93.45	413	4.05	717	-
spruceA0P07	-	47.50	-93.45	413	4.05	717	-
spruceT0P06	-	47.50	-93.45	410	4.05	717	-
spruceT0P19E	-	47.50	-93.45	410	4.05	717	-
thompsonfarm2N	-	43.10	-70.95	23	8.7	1247	Research at the Thompson Farm Observatory is supported by NH EPSCoR with support from the National Science Foundation's Research Infrastructure Improvement Award (#EPS 1101245) and by the NH Agricultural Experiment Station/USDA NIFA (Hatch project #1006997).
turkeypointenf02	CA-TP1	42.66	-80.55	194	8.85	1019	Research at this site was funded by the Natural Sciences and Engineering Research Council (NSERC) of Canada, Canadian Foundation of Innovation (CFI), Ontario Ministry of Research and Innovation (MRI) and Ontario Ministry of Environment, Conservation and Parks (MECP). Support from Ontario Ministry of Natural Resources and Forestry (OMNRF), St Williams Conservation Reserve Community Council (SWCRCC), Long Point Conservation Authority (LPRCA), Whitside family and McMaster University is also acknowledged.
turkeypointenf39	CA-TP4	42.71	-80.35	232	8.65	1015	Research at this site was funded by the Natural Sciences and Engineering Research Council (NSERC) of Canada, Canadian Foundation of Innovation (CFI), Ontario Ministry of Research and Innovation (MRI) and Ontario Ministry of Environment, Conservation and Parks (MECP). Support from Ontario Ministry of Natural Resources and Forestry (OMNRF), St Williams Conservation Reserve Community Council (SWCRCC), Long Point Conservation Authority (LPRCA), Whitside family and McMaster University is also acknowledged.
turkeypointenf74	CA-TP3	42.70	-80.34	216	8.65	1013	Research at this site was funded by the Natural Sciences and Engineering Research Council (NSERC) of Canada, Canadian Foundation of Innovation (CFI), Ontario Ministry of Research and Innovation (MRI) and Ontario Ministry of Environment, Conservation and Parks (MECP). Support from Ontario Ministry of Natural Resources and Forestry (OMNRF), St Williams Conservation Reserve Community Council (SWCRCC), Long Point Conservation Authority (LPRCA), Whitside family and McMaster University is also acknowledged.
umichbiological	US-UMB	45.55	-84.71	230	6.35	846	Primary support for the University of Michigan AmeriFlux Core Site(US-UMB) provided by the Department of Energy Office of Science. Infrastructure support provided by the University of Michigan Biological Station.
usmpj	US-Mpj	34.43	-106.25	2126	10.5	421	-
windriver	US-Wrc	45.82	-121.95	371	9.55	2264	Data and logistical support were provided by the US Forest Service Pacific Northwest Research Station and the University of Washington

Table S3 Evaluation of correlation of start-of-season (SOS) and end-of-season (EOS) transition dates, derived from PhenoCam imagery, with corresponding dates derived from tower-based estimates of gross primary productivity (GPP). We compared two different indices of canopy color, *G*cc (green chromatic coordinate) and GRVI (green-red vegetation index). We aggregated data from multiple images recorded over a 3-day period to a single value using different aggregation statistics ("Aggr. Stat.": mean, median, 75th percentile, and 90th percentile), following Sonnentag *et al.*, 2012. We extracted transition dates from the 3-day data using different thresholds ("Δ Thresh.": 10 %, 25 %, and 50 %) of the seasonal amplitude of each index (see Richardson *et al.*, 2018a). We used Type II (geometric mean) regression to quantify the relationship between PhenoCam-based and GPP-based transition dates, where m and b are the slope and intercept of the fitted line, R^2 is the coefficient of determination, and RMSE is the root mean squared error of the fitted line. Methods are ranked by Pearson's correlation separately for SOS and EOS, and by the mean correlation across both SOS and EOS.

	**Aggr.**	**Δ Thresh.**	**SOS**				**EOS**				**Rank by *r***		**Mean *r***	
**Index**	**Stat.**		**m**	**b**	**R^2**	**RMSE**	**m**	**b**	**R^2**	**RMSE**	**SOS**	**EOS**	**mean**	**rank**
*G*cc	90 pctl	25 %	0.53	45.7	0.49	6.05	0.84	50.4	0.54	10.61	1	10	0.72	1
GRVI	mean	50 %	0.74	50.3	0.48	10.11	0.74	60.9	0.55	11.04	3	9	0.72	2
*G*cc	mean	10 %	0.37	51.8	0.41	6.93	0.75	86.4	0.62	10.76	9	3	0.71	3
*G*cc	75 pctl	10 %	0.35	54.1	0.41	6.66	0.69	105.5	0.59	10.87	7	7	0.70	4
*G*cc	75 pctl	25 %	0.50	48.4	0.39	6.39	0.86	42.7	0.61	10.38	11	5	0.70	5
GRVI	mean	25 %	0.97	19.1	0.38	13.23	1.02	-17.7	0.59	13.05	12	6	0.69	6
*G*cc	median	10 %	0.36	53.2	0.35	7.28	0.75	86.9	0.63	10.91	14	2	0.69	7
*G*cc	median	10 %	0.37	52.2	0.47	6.73	0.70	103.7	0.50	11.71	4	14	0.69	8
*G*cc	median	25 %	0.53	45.4	0.34	7.11	0.92	24.6	0.65	10.50	16	1	0.69	9
GRVI	median	50 %	0.75	49.6	0.48	10.14	0.68	75.7	0.46	10.29	2	19	0.69	10
*G*cc	mean	25 %	0.55	42.3	0.34	7.38	0.94	19.8	0.62	10.65	15	4	0.69	11
GRVI	median	25 %	0.98	17.8	0.36	13.64	0.95	5.4	0.53	12.52	13	12	0.67	12
GRVI	90 pctl	25 %	1.01	15.2	0.41	13.45	0.86	35.1	0.47	12.72	10	17	0.66	13

GRVI	75 pctl	25 %	0.79	37.2	0.41	10.47	0.93	9.8	0.45	13.74	8	21	0.65	14
GRVI	90 pctl	50 %	0.71	55.3	0.42	10.40	0.61	99.0	0.43	10.37	5	22	0.65	15
GRVI	75 pctl	50 %	0.69	57.3	0.41	9.36	0.65	85.3	0.41	11.02	6	24	0.64	16
GRVI	mean	10 %	0.71	40.8	0.25	16.14	1.00	-4.8	0.55	16.36	17	8	0.62	17
GRVI	median	10 %	0.74	38.9	0.24	16.78	0.93	15.9	0.52	15.30	18	13	0.61	18
GRVI	75 pctl	10 %	0.56	53.8	0.24	12.71	0.95	9.5	0.47	16.58	19	16	0.59	19
GRVI	90 pctl	10v	0.86	27.1	0.22	17.41	0.85	45.3	0.46	15.65	20	20	0.57	20
*G*cc	mean	50 %	0.55	55.5	0.14	9.59	0.80	53.0	0.54	11.31	23	11	0.56	21
*G*cc	75 pctl	50 %	0.49	63.7	0.17	8.57	0.73	73.2	0.46	11.87	22	18	0.55	22
*G*cc	90 pctl	50 %	0.47	66.4	0.19	8.04	0.69	86.2	0.43	11.30	21	23	0.55	23
*G*cc	median	50 %	0.52	59.2	0.14	9.43	0.79	55.1	0.49	12.83	24	15	0.54	24

Table S4 List of the fitted model parameters for the model presented in Eq. 6 -8.

site	ROI ID	G_{max}	G_{min}	θ_1	θ_2	ρ_1	ρ_2	D	σ
canadaOBS	1000	0.3867	0.3262	-7.67	4.94	9.80E-05	2.34E-03	183	0.0073
harvardbarn	1000	0.3830	0.3221	2.79	4.95	3.67E-04	3.97E-04	129	0.0091
harvardbarn2	1000	0.4341	0.3479	-0.56	4.80	2.32E-04	4.15E-04	132	0.0131
harvardhemlock	2000	0.4190	0.3743	0.33	4.91	6.69E-05	5.51E-04	157	0.0065
harvardhemlock2	1000	0.3932	0.3267	-3.37	4.77	5.89E-05	3.71E-04	168	0.0084
howland1	3000	0.3897	0.3540	-0.45	4.96	9.35E-05	3.21E-04	151	0.0060
huyckpreserveny	1000	0.4113	0.3743	-8.28	4.67	4.61E-05	1.61E-04	126	0.0064
laclaflamme	1000	0.4059	0.3291	1.24	-7.53	3.76E-04	2.97E-01	148	0.0110
laurentides	1000	0.4277	0.3492	-2.45	4.94	1.94E-04	8.10E-04	147	0.0084
missouriozarks	1000	0.4077	0.3338	-0.95	4.89	9.60E-05	1.57E-03	145	0.0124
niwot2	1000	0.4805	0.4228	-9.63	4.83	6.14E-05	1.70E-04	155	0.0098
niwot3	1000	0.3963	0.3536	-0.37	4.91	1.20E-04	3.87E-04	179	0.0057
niwot5	1000	0.4113	0.3675	-7.79	4.82	7.13E-05	2.48E-04	190	0.0062
oregonMP	1000	0.3714	0.3366	-5.15	4.96	4.18E-05	2.64E-04	163	0.0064
oregonYP	2000	0.4113	0.3651	-8.90	4.92	3.38E-05	1.68E-04	142	0.0056
spruceA0EMI	1000	0.3602	0.3125	-3.77	4.60	7.30E-05	8.97E-04	154	0.0058
spruceA0P07	1000	0.4137	0.3410	-8.76	4.36	9.92E-05	1.08E-03	144	0.0078
spruceT0P06	1000	0.4035	0.3465	-7.49	4.80	7.17E-05	8.73E-04	143	0.0073
spruceT0P19E	1000	0.3899	0.3348	-7.92	4.81	6.46E-05	7.86E-04	145	0.0074
thompsonfarm2N	2000	0.3460	0.3168	2.81	4.95	1.15E-04	2.65E-04	133	0.0041
turkeypointenf02	1000	0.3594	0.3191	0.29	4.95	1.64E-04	5.46E-04	133	0.0062
turkeypointenf39	1000	0.3666	0.3200	-3.44	4.95	1.00E-04	7.10E-04	143	0.0085
turkeypointenf74	1000	0.4123	0.3368	-9.00	4.93	7.13E-05	8.75E-04	149	0.0113
umichbiological	1000	0.4814	0.4289	-2.35	4.89	2.60E-04	2.74E-04	121	0.0126
umichbiological	2000	0.4065	0.3155	-0.83	4.91	4.02E-04	6.32E-04	133	0.0135
usmpj	1000	0.3653	0.3356	-6.24	4.78	1.70E-05	3.18E-04	163	0.0043
windriver	1000	0.3979	0.3653	-8.63	4.86	2.54E-05	3.85E-04	152	0.0056
**Mean**	-	0.4011	0.3465	-3.94	4.39	1.27E-04	1.15E-02	149	0.0080
**CV**	-	0.079	0.0843	-1.024	0.543	0.858	4.923	0.113	0.337

Reference:

Arain MA. **2003a**. AmeriFlux CA-TP1 Ontario - Turkey Point 2002 Plantation White Pine. *Dataset*: https://doi.org/10.17190/AMF/1246009.

Arain MA. **2003b**. AmeriFlux CA-TP3 Ontario - Turkey Point 1974 Plantation White Pine. *Dataset*: https://doi.org/10.17190/AMF/1246011.

Arain MA. **2003c**. AmeriFlux CA-TP4 Ontario - Turkey Point 1939 Plantation White Pine. *Dataset*: https://doi.org/10.17190/AMF/1246012.

Arain MA, Restrepo-Coupe N. **2005**. Net ecosystem production in a temperate pine plantation in southeastern Canada. *Agricultural and Forest Meteorology* **128**: 223–241.

Bergeron O, Margolis HA, Black TA, Coursolle C, Dunn AL, Barr AG, Wofsy SC. **2007**. Comparison of carbon dioxide fluxes over three boreal black spruce forests in Canada. *Global Change Biology* **13**: 89–107.

Blanken PD, Monson RK, Burns SP, Bowling DR, Turnipseed AA. **1998**. AmeriFlux US-NR1 Niwot Ridge Forest (LTER NWT1). *Dataset*: https://doi.org/10.17190/AMF/1246088.

Burns SP, Blanken PD, Turnipseed AA, Hu J, Monson RK. **2015**. The influence of warm-season precipitation on the diel cycle of the surface energy balance and carbon dioxide at a Colorado subalpine forest site. *Biogeosciences* **12**: 7349–7377.

Chan FCC, Altaf Arain M, Khomik M, Brodeur JJ, Peichl M, Restrepo-Coupe N, Thorne R, Beamesderfer E, McKenzie S, Xu B, *et al.* 2018. Carbon, water and energy exchange dynamics of a young pine plantation forest during the initial fourteen years of

growth. *Forest Ecology and Management* **410**: 12–26.

Hollinger D. **1996**. AmeriFlux US-Ho1 Howland Forest (main tower). *Dataset*: https://doi.org/10.17190/AMF/1246061.

Hollinger DY, Goltz SM, Davidson EA, Lee JT, Tu K, Valentine HT. **1999**. Seasonal patterns and environmental control of carbon dioxide and water vapour exchange in an ecotonal boreal forest. *Global Change Biology* **5**: 891–902.

Jassal RS, Black TA, Spittlehouse DL, Brümmer C, Nesic Z. **2009**. Evapotranspiration and water use efficiency in different-aged Pacific Northwest Douglas-fir stands. *Agricultural and Forest Meteorology* **149**: 1168–1178.

Kwon H, Law BE, Thomas CK, Johnson BG. **2018**. The influence of hydrological variability on inherent water use efficiency in forests of contrasting composition, age, and precipitation regimes in the Pacific Northwest. *Agricultural and Forest Meteorology* **249**: 488–500.

Law B. **2002**. AmeriFlux US-Me2 Metolius mature ponderosa pine. *Dataset*: https://doi.org/10.17190/AMF/1246076.

Law B. **2010**. AmeriFlux US-Me6 Metolius Young Pine Burn. *Dataset*: https://doi.org/10.17190/AMF/1246128.

Law BE, Berner LT. **2015**. NACP TERRA-PNW: Forest Plant Traits, NPP, Biomass, and Soil Properties, 1999-2014.

Liu P, Black TA, Jassal RS, Zha T, Nesic Z, Barr AG, Helgason WD, Jia X, Tian Y, Stephens JJ, *et al.* 2019. Divergent long-term trends and interannual variation in ecosystem resource use efficiencies of a southern boreal old black spruce forest 1999–2017. *Global Change Biology* **25**: 3056–3069.

Margolis HA. **2003**. AmeriFlux CA-Qfo Quebec - Eastern Boreal, Mature Black Spruce. *Dataset*: https://doi.org/10.17190/AMF/1246829.

Martin T. **2000**. AmeriFlux US-SP1 Slashpine-Austin Cary- 65yrs nat regen. *Dataset*: https://doi.org/10.17190/AMF/1246100.

Peichl M, Brodeur JJ, Khomik M, Arain MA. **2010**. Biometric and eddy-covariance based estimates of carbon fluxes in an age-sequence of temperate pine forests. *Agricultural and Forest Meteorology* **150**: 952–965.

Richardson AD, Hollinger DY, Shoemaker JK, Hughes H, Savage K, Davidson EA. **2019**. Six years of ecosystem-atmosphere greenhouse gas fluxes measured in a sub-boreal forest. *Scientific Data* **6**: 117.

Richardson AD, Hufkens K, Milliman T, Aubrecht DM, Chen M, Gray JM, Johnston MR, Keenan TF, Klosterman ST, Kosmala M, *et al.* 2018. Tracking vegetation phenology across diverse North American biomes using PhenoCam imagery. *Scientific Data* **5**: 180028.

Ruehr NK, Martin JG, Law BE. **2012**. Effects of water availability on carbon and water exchange in a young ponderosa pine forest: Above- and belowground responses. *Agricultural and Forest Meteorology* **164**: 136–148.

Sonnentag O, Hufkens K, Teshera-Sterne C, Young AM, Friedl M, Braswell BH, Milliman T, O’Keefe J, Richardson AD. **2012**. Digital repeat photography for phenological research in forest ecosystems. *Agricultural and Forest Meteorology* **152**: 159–177.
